# Smoking Cessation and the Microbiome in Induced Sputum Samples from Cigarette Smoking Asthma Patients

**DOI:** 10.1371/journal.pone.0158622

**Published:** 2016-07-08

**Authors:** Christian Munck, Jens Helby, Christian G. Westergaard, Celeste Porsbjerg, Vibeke Backer, Lars H. Hansen

**Affiliations:** 1 Technical University of Denmark, The Novo Nordisk Foundation Center for Biosustainability, Hørsholm, Denmark; 2 Department of Clinical Biochemistry, Herlev and Gentofte Hospital, Copenhagen University Hospital, Herlev, Denmark; 3 Respiratory Research Unit, Bispebjerg University Hospital, Copenhagen, Denmark; 4 Aarhus University, Department of Environmental Science, Roskilde, Denmark; University of Athens, GREECE

## Abstract

Asthma is a common disease causing cough, wheezing and shortness of breath. It has been shown that the lung microbiota in asthma patients is different from the lung microbiota in healthy controls suggesting that a connection between asthma and the lung microbiome exists. Individuals with asthma who are also tobacco smokers experience more severe asthma symptoms and smoking cessation is associated with improved asthma control. In the present study we investigated if smoking cessation in asthma patients is associated with a change in the bacterial community in the lungs, examined using induced sputum. We found that while tobacco smokers with asthma have a greater bacterial diversity in the induced sputum compared to non-smoking healthy controls, smoking cessation does not lead to a change in the microbial diversity.

## Introduction

Traditionally the lungs have been considered a sterile environment. However, with the advent of culture independent techniques to study bacterial populations it has been shown that the lungs harbor a specific bacterial community [1]. Several studies have used culture independent techniques to characterize the bacterial community in the lungs of different individuals including tobacco smokers, asthma patients, patients with chronic obstructive pulmonary disease (COPD) and healthy controls [2–5].

For individuals with asthma it has been shown that the bacterial community in the lungs is different from the community in healthy controls [2,6–8]. Specifically, it has been found that the bacterial diversity in the lungs of asthma patients is greater than the bacterial diversity in the lungs of healthy controls and that the diversity correlates with bronchial hyper responsiveness [6,8].

Studies of the lung microbiota in tobacco smoking individuals who are otherwise healthy indicate that smoking is not associated with changes in the bacterial composition compared to individuals who are non-smokers [4]. In contrast, studies of the oral microbiome of tobacco smokers found that smoking is associated with changes in the oral microbiome compared to non-smokers [4,5]. However, the extent to which tobacco smoking is associated with changes in the microbiome in individuals with an underlying pulmonary disease is not well known. In asthma patients, tobacco smoking is associated with increased asthma symptom severity as well as increased risk of asthma-exacerbations and death [9,10]. Furthermore, tobacco smokers with asthma have a significantly increased risk of pneumonia compared to healthy controls [11]. Despite this, cigarette smoking is as common in people with asthma as in healthy individuals [9].

We have recently shown that cigarette smoking asthma patients who quit smoking experience an overall increase in asthma control as well as reduced airway hyper responsiveness [12,13]. Whether the smoking cessation is associated with changes in the bacterial communities in the lungs is not known. The aim of the present study was to use culture independent techniques to analyze the bacterial community in sputum samples collected longitudinally from cigarette smoking asthma patients who attempt smoking cessation. Specifically, we tested the hypothesis that smoking cessation is associated with changes in bacterial diversity.

## Material and Methods

### Study group

The 44 asthma patients enrolled in this study were part of a randomized clinical trial on the effect of varenicline versus placebo on cigarette smoking cessation in individuals with asthma [13]. The study design of the randomized trial, including detailed inclusion and exclusion criteria, have been described previously [12]. In summary, 52 cigarette smoking asthma patients who had not, within the last 3 months, experienced lower respiratory tract infections or received treatment with inhaled or systemic corticosteroids were randomized 1:1 to receive either varenicline or placebo for 12 weeks. Induced sputum samples for microbiological analysis were collected at two time points, week 0 and week 12. Among the 52 randomized patients, 8 patients were excluded from the present study since they either dropped out or did not complete the collection of induced sputum at both week 0 and week 12. The cigarette smoking status of the patients was evaluated through interview and CO breath test at week 0, week 6 and week 12. In addition to the patient samples, baseline control samples were also collected from a group of 20 non-smoking, non-asthmatic healthy controls, recruited through advertisement. The study was approved by The Copenhagen County Ethical Committee (www.regionh.dk/vek, ref. H-2-2010-140) and all asthma patients and control subjects provided written, informed consent. The randomized trial was registered at clinicaltrial.gov (NTC: NCT02233231) under the identifier: "Young smoking asthmatics".

### Sputum sample collection

Prior to collecting the induced sputum sample, participants washed their mouth with water to reduce oral contamination. For each patient, 1–2 ml induced sputum sample was collected and transferred directly to a sterile 15 ml tube containing 4 ml lysogeny broth (LB) medium with 27% glycerol, as we generally experience that LB preserves cultivability. The tubes were kept on ice and immediately frozen at -20°C and transferred to -80°C. Further details on the collection of induced sputum in this study have been published previously [13,14].

### DNA extraction

The samples were thawed at room temperature and 5 ml 0.2% dithiothreitol (DTT) solution was mixed with the sample and incubated for 10–15 minutes at room temperature to dissolve viscous sample parts. Next, the tubes were centrifuged at 7500 g for 5 minutes and the supernatant was removed. The pellet was heat inactivated for 5 minutes at 95°C in order to kill potential pathogens. The pellet was dissolved in 500 μl bead solution from the PowerLyzer™ PowerSoil® DNA Isolation Kit (MOBIO) and DNA was extracted according to the manufacture’s protocol using the Fast Prep (MPBIO) homogenizer at speed 6 for 40 seconds. The DNA was eluted in 50 μl elution buffer and stored at -20°C.

### 16S sequencing

For each of the 88 patient samples and the 20 control samples DNA was extracted and analyzed by 16S rRNA gene amplicon sequencing using the 454 FLX platform (Roche). All 16S libraries were constructed by amplifying the V3-V4 region of the 16S rrna gene using the 341F primer CCTAYGGGRBGCASCAG and the 806R primer GGACTACNNGGGTATCTAAT [15]. PCR was performed using the AccuPrime PCR mixture (Invitrogen) with 5 μl template in a total volume of 25 μl using the following cycling conditions: 94°C for 2 min followed by 30 cycles of 94°C for 20 sec, 56°C for 30 sec, 68°C for 40 sec and final extension at 68°C for 5 min. After PCR the 16S V3-V4 band was gel extracted using the Montage gel extraction kit (Millipore). For PCR reactions that did not result in a visible band (8 at week 0, 4 at week 12 and 7 controls) the template DNA concentrations were measured using a Qubit (Invitrogen), in all cases the DNA concentration was less than 1 ng/μl. These samples were successfully amplified in a new PCR attempt using 10 μl template and 35 cycles. In parallel with all 16S rRNA gene amplifications control reactions with no template were run, which gave no PCR product. Next all samples were barcoded in a second 15 cycles PCR reaction using 2 μl template from the first PCR reaction. The DNA concentration of the barcoded samples was measured with Qubit and the samples were pooled to equal quantities and sequenced using the 454 FLX platform (Roche).

### Data analysis

Quality filtering, read trimming and OTU clustering was done according to the UPARSE pipeline [16]. Briefly, reads were trimmed to 400 bp and singletons or reads with expected error greater than 0.5 were filtered out. OTUs were defined using an identity cut-off of 97% as this generally approximates the difference in 16S sequences between bacterial species [17]. The OTU clusters were filtered for chimeras using the Gold database [13]. Taxonomy was assigned using the rdp classifier in QIIME and greengenes 13_8 reference database (greengenes.lbl.gov) trimmed to sequences with complete taxonomic path [18,19]. Subsequent data analysis was performed in R using the phyloseq package to calculate diversity measures and in Stata (StataCorp LP) version 13.1 to perform statistical analysis [20].

For comparisons between asthma patients and controls and for comparisons at baseline between cigarette quitting and non-quitting asthma patients, unpaired t-test and Mann-Whitney U-test was applied for continuous variables with parametric and non-parametric distributions respectively. For categorical variables, Pearson's chi-squared test was applied. When comparing differences in change from week 0 to week 12 between quitters and non-quitters or between the varenicline and the placebo group, unpaired t-test and Mann-Whitney U-test using the paired differences (change from week 0 to week 12) was applied for paired differences with parametric and non-parametric distributions respectively. In the comparison of the quitters at week 0 to week 12, a paired t-test and a Wilcoxon Signed-Rank Test was used for continuous variables with parametric and non-parametric distributions respectively. The trimmed greengenes database includes 277 different bacterial families. Since our analysis of differences in abundance of specific families was exploratory and not driven by pre-specified hypotheses, all analysis regarding specific families were adjusted for 277 multiple comparisons using the Bonferroni method.

## Results

### Baseline profile

Of the 44 included asthma patients, 25 had quit cigarette smoking at week 12 while 19 had not. Of the 25 who quit cigarette smoking 24 had already stopped at week 6, highlighting that smoking cessation for the most part occurred early in the intervention. Detailed baseline characteristics for the 44 patients according to smoking status at week 12 has been published previously [12] and are summarized along with baseline characteristics for the 20 healthy control subjects in Table 1. There were no differences at baseline with respect to age, sex, spirometric variables or cumulative smoking in pack-years between the patients who quit smoking and those who did not (Table 1).

**Table 1:** Baseline characteristics for controls and asthma patients according to cigarette smoking status at week 12.

Parameters	Controls	Quitters	Non-quitters	P*
Individuals, n	20	25	19	-
Age, years	25 ± 3	32 ± 6	32 ± 6	0.84
Male sex, %	10 (50%)	15 (60%)	13 (68%)	0.57
FEV1, % of predicted	101 ± 9	84 ± 18	83 ± 16	0.86
FVC, % of predicted	104 ± 11	97 ± 14	96 ± 12	0.81
FEV1/FVC, %	83 ± 7	72 ± 10	73 ± 10	0.89
Cumulative smoking, pack-years	0	16 ± 5	16 ± 4	0.91

Mean ± SD are shown for continuous variables while number (%) is shown for categorical variables.

*P-value for cigarette quitters versus non-quitters using unpaired t-test and Pearson's chi-squared test for continuous and categorical variables respectively.

In total, 88 16S rRNA gene amplicons from the asthma patients and 20 amplicons from the healthy controls were sequenced. On average each amplicon contained 7844 sequences (S1 Fig). Clustering of these sequences at 97% identity resulted in a total of 476 different operational taxonomic units (OTUs) with each sample on average containing 120 OTUs (sd = 25) (S2 Fig). Rarefaction curve analysis showed that the majority of the OTUs in each sample were captured, indicating that the sequencing depth was sufficient to uncover most of the diversity in the sample (S3 Fig).

**Fig 1:**
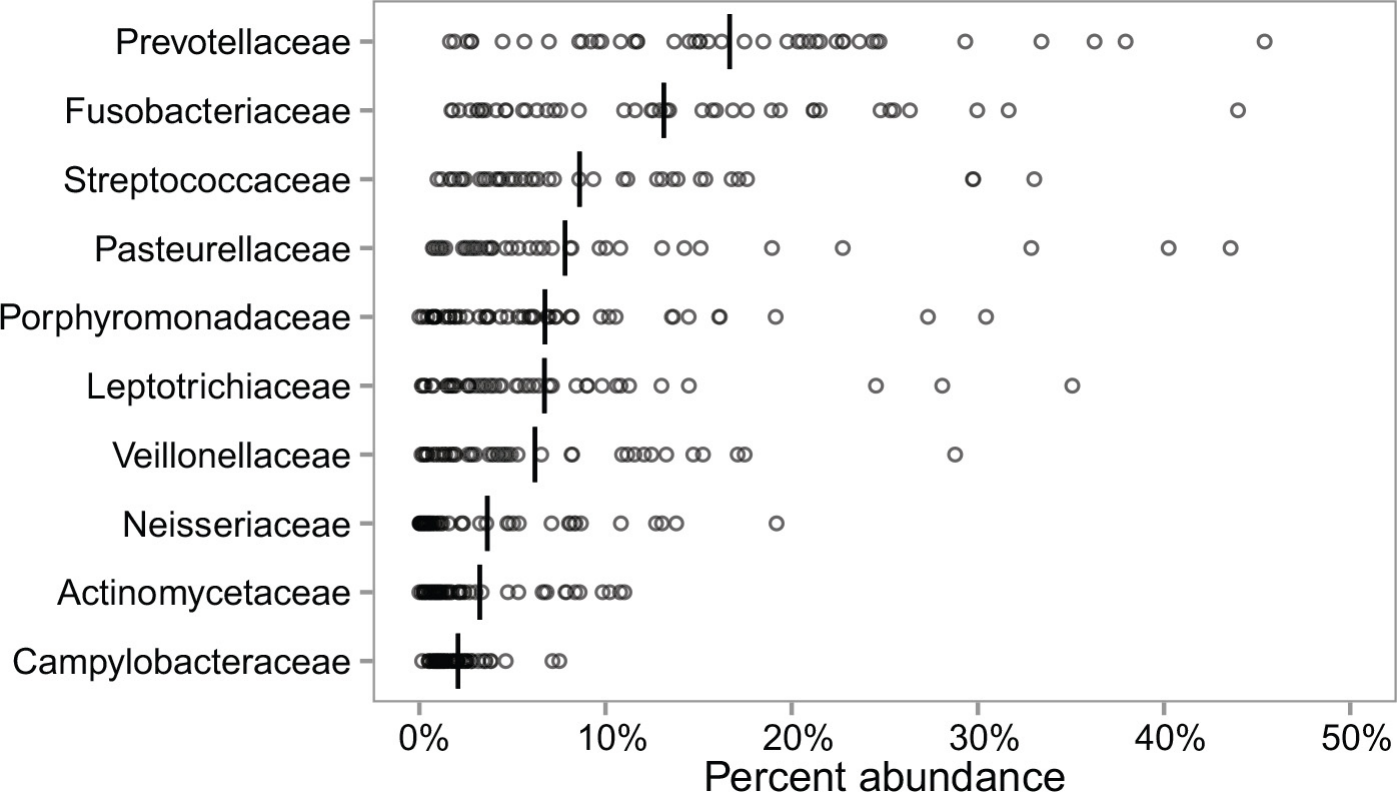
Most abundant bacterial families at week 0. The distribution of the ten most abundant families in the week 0 asthma patient samples. For each sample (n = 44) the percent of the reads belonging to the specified family is shown. The vertical crossbar marks the mean of the distribution.

At week 0 the samples from the asthma patients contained a total of 378 different OTUs with each sample on average containing 123 OTUs (sd = 25) (S2 Fig). Taxonomy could be assigned to the level of genus or lower to 157 of the 378 OTUs (42%) and to family or lower to 267 (71%) of the OTUs. As the taxonomic resolution of the relatively short (400 bp) 16S rRNA gene sequences is limited [14] the taxonomic profiles of the samples are presented at the family level. The ten most abundant families, measured as the mean of the relative abundance in each sample, were Prevotellaceae (17%), Fusobacteriaceae (13%), Streptococcaceae (9%), Pasteurellaceae (8%), Porphyromonadaceae (7%), Leptotrichiaceae (7%), Veillonellaceae (6%), Neisseriaceae (4%), Actinomycetaceae (3%) and Campylobacteraceae (2%) (Fig 1). These families have all been reported in previous studies of the lung microbiome and are also common members of the oral microbiome [3,4,21]. As the difference between the lung and the oral microbiome to a large extend is abundance driven, as opposed to species driven, it is challenging to filter-out the oral flora from the lung microbiome [4,21].

**Fig 2:**
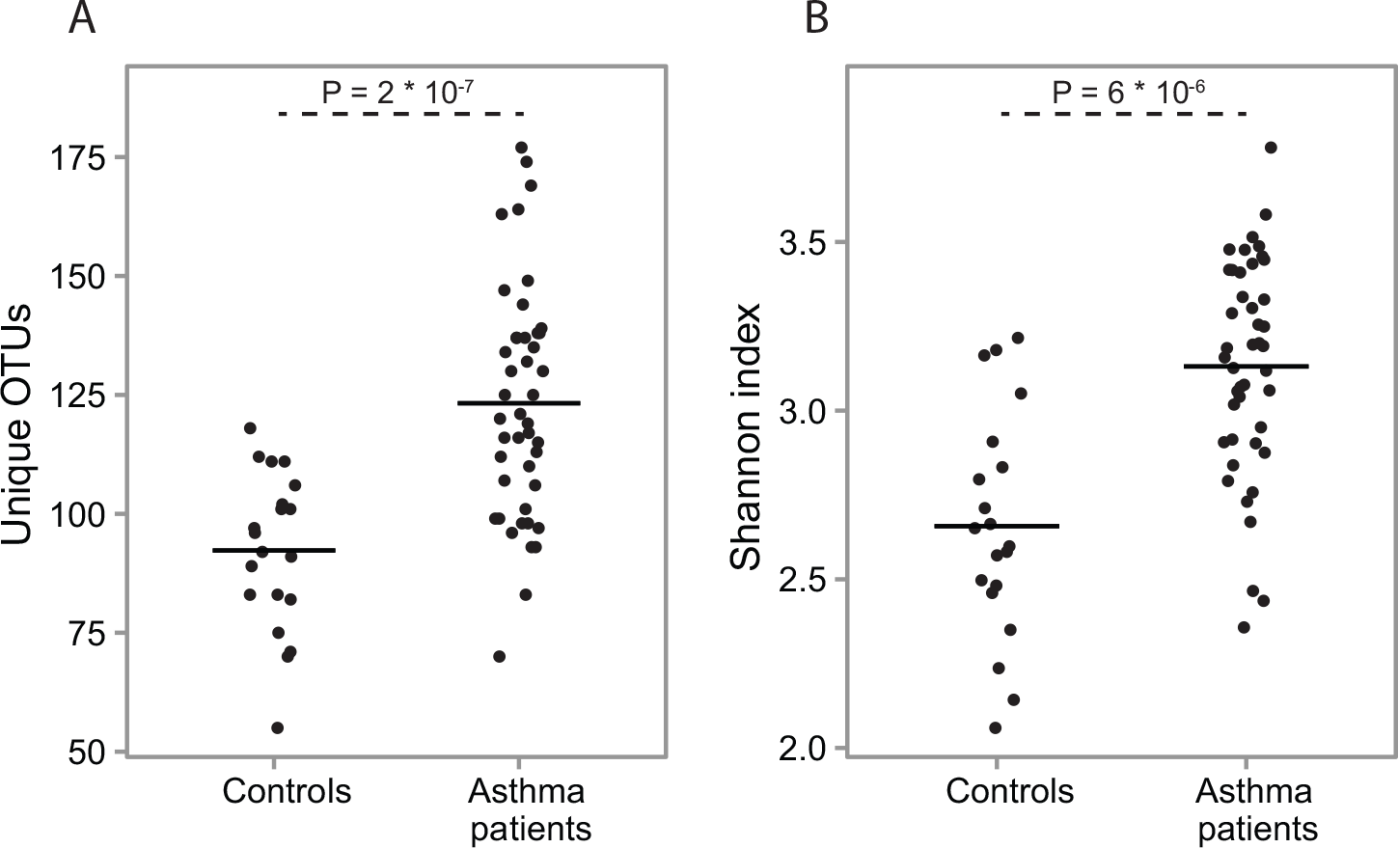
Diversity at week 0. A) The distribution of unique OTUs in the asthma patients and the healthy controls. The solid crossbars mark the mean of each distribution. The samples from the asthma patients contain significantly more unique OTUs compared to samples from the healthy controls (unpaired t-test). B) The Shannon diversity index in samples from the asthma patients and the healthy controls. The solid crossbars mark the mean of each distribution. Samples from the asthma patients contain a significantly higher bacterial diversity than samples from the healthy controls (unpaired t-test).

Comparisons of the bacterial diversity in the week 0 patient samples to the control samples showed that both the number of OTUs and the Shannon index were significantly higher in the patient samples relative to the healthy controls (P = 2 * 10^−7^ and P = 6 * 10^−6^, respectively, unpaired t-test) (Fig 2A and 2B). While the number of OTUs is a simple unweighted measure of diversity the Shannon index takes into account the relative abundances of each OTU in a sample [22].

**Fig 3:**
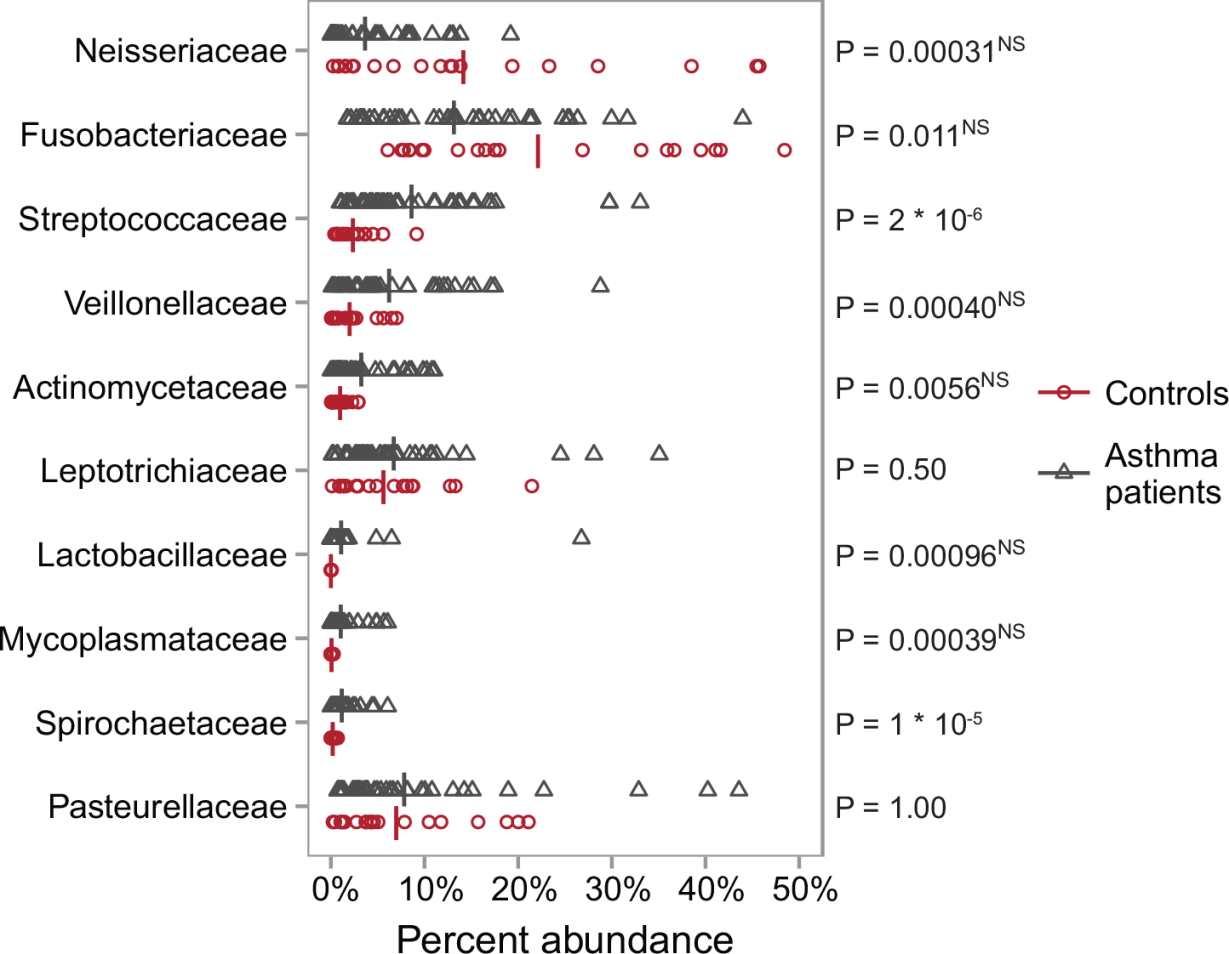
Most different bacterial families at week 0. The ten families with the biggest absolute difference in mean abundance in samples from asthma patients relative to samples from healthy controls at week 0. The crossbar marks the mean of each distribution. ^NS^ P-value not significant at the 0.05 confidence level after correction for 277 multiple comparisons using the Bonferroni-method (Mann-Whitney U test, required P-value < 1.81 * 10^−4^ = 0.05 / 277).

To further explore the difference between the controls and the patient group we identified the ten bacterial families with the largest absolute difference in mean abundance between the two groups (Fig 3). Two of these families, Streptococcaceae (P = 2 * 10^−6^, Mann-Whitney U-test) and Spirochaetaceae (P = 1 * 10^−5^) were significantly more abundant in the asthma patients compared to the controls after correction for 277 multiple comparisons using the Bonferroni-method (required P-value < 1.81 * 10^−4^ = 0.05 / 277 unique OTUs in the database). However, as these families contain several species commonly found in the oral and lung flora, including opportunistic pathogens, it is difficult to comment on the clinical implications of this finding.

### Changes associated with smoking cessation

**Fig 4:**
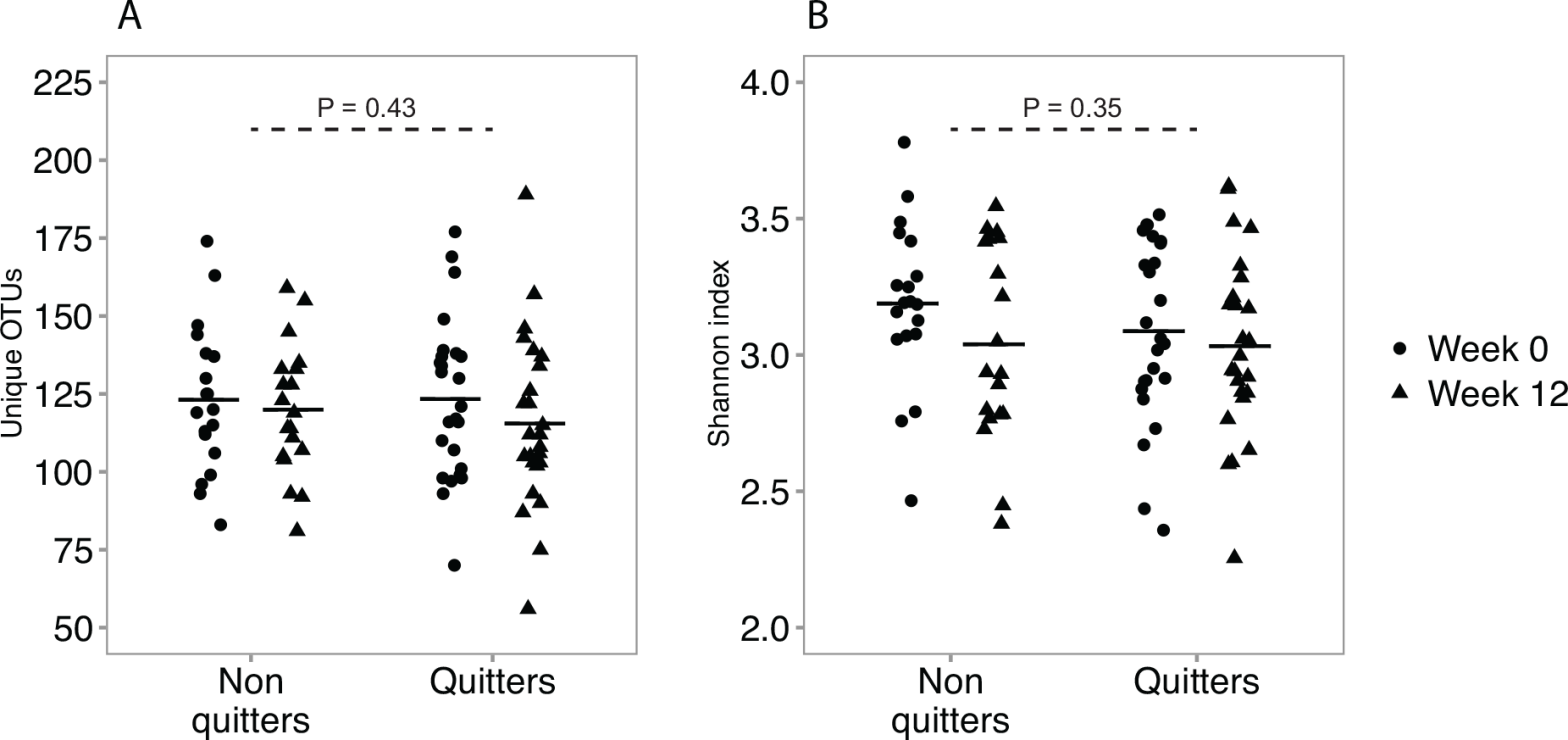
Bacterial diversity at week 0 and week 12. A) The number of unique OTUs for the non-quitters and the quitters at week 0 and week 12. The solid crossbars mark the mean of each distribution. When comparing the change (i.e. from week 0 to week 12) in the non-quitter group to the change in the quitter group no significant difference was found (P = 0.43, unpaired t-test of the paired differences). B) The Shannon diversity index for the non-quitters and quitters at week 0 and week 12. The solid crossbars mark the mean of each distribution. When comparing the change (i.e. from week 0 to week 12) in the non-quitter group to the change in the quitter group no significant difference was found (P = 0.35, unpaired t-test of paired differences).

The longitudinal design of our study allowed us to compare changes in the bacterial communities in the lungs within the same patients before and after cigarette smoking cessation. This helped eliminate inter-patient variation, which could otherwise mask the changes. At week 12, 25 patients had quit cigarette smoking while 19 were still smoking (Table 1). When comparing quitters and non-quitters there was no significant difference in the mean change (i.e. from week 0 to week 12) in the number of unique OTUs (P = 0.43, unpaired t-test of the paired differences) or the Shannon index (P = 0.35, unpaired t-test of the paired differences) (Fig 4A and 4B). Similarly, when comparing the change in the number of OTUs and the Shannon index from week 0 to week 12 according to randomization (i.e. varenicline vs. placebo) we found no significant differences between the two groups (P = 0.45 and P = 0.61, respectively, unpaired t-test of the paired differences S4 Fig).

As all patients attempted smoking cessation we cannot rule out that the non-quitter group displayed reduced smoking frequency potentially masking the effect of smoking cessation on the diversity in the microbiome. To reduce this impact we investigated if there was any change in the bacterial diversity within the quitter group alone. We found no significant change in the number of OTUs or the Shannon index from week 0 to week 12 (P = 0.08 and P = 0.38, respectively, paired t-test)(S5 Fig). While this comparison cannot account for biological variation or non-intervention effects, it does highlight that when looking at the quitters alone, there is no obvious association between quitting cigarette smoking and the microbiome diversity. This suggests, that although cigarette smoking asthma patients who quit smoking experience improved asthma control and reduced airway hyper responsiveness this is not, after a cigarette-free period of at least 6 weeks, accompanied by a change in the bacterial diversity of the lung microbiome.

**Fig 5:**
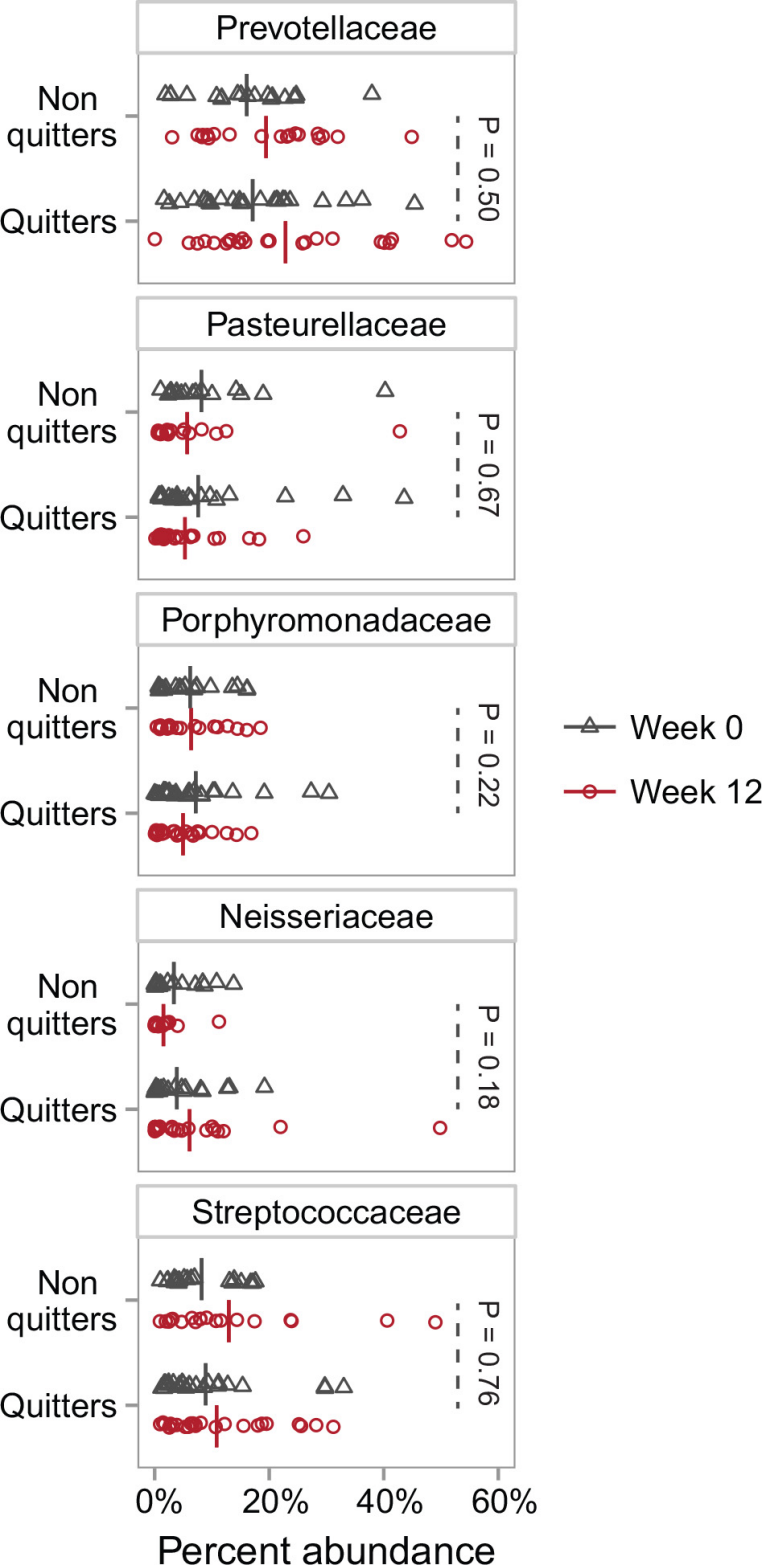
Families that differed most after attempting smoking cessation. The five families, within the group of patients that quit cigarette smoking, with the largest mean change (i.e. from week 0 to week 12) in relative abundance. When comparing the change in abundance (i.e. from week 0 to week 12) in the quitter group to the change in the non-quitter group there was no significant differences (Mann-Whitney U-test of the within-patient change in the quitter group compared to the within-patient change in the non-quitter group).

To explore if specific bacterial families were affected by smoking cessation we identified the five families with the largest mean change in the quitter group. For these families we compared the change (i.e. from week 0 to week 12) in the quitter group to the change in the non-quitter group (Mann-Whitney U-test of the within-patient change in the quitter group compared to the within-patient change in the non-quitter group) (Fig 5). For each family, except Neisseriaceae and Porphyromonadaceae, the change in abundance from week 0 to week 12 was in the same direction for both the quitter and the non-quitter group (Fig 5). As all samples were processed in parallel this uniform shift is not likely to be caused by differences in laboratory procedures. None of the changes in family abundance were significantly different between the quitter group and the non-quitter group (Fig 5). This indicates that within the investigated timespan smoking cessation was not associated with major changes in specific bacterial families in the sputum microbiome. To investigate if a potential reduced smoking frequency in the non-quitter group could mask a difference in the five families, we compared the family abundances at week 0 to the abundance at week 12 for the quitter group. In this comparison we found no significant difference in any of the five families (Wilcoxon Signed-Rank Test, data not shown).

## Discussion

We have recently shown that cigarette smoking cessation is associated with improvements in asthma control in cigarette smoking asthma patients [12]. In the present study we investigated if smoking cessation was also associated with changes in the bacterial diversity of the sputum microbiome. We investigated the microbiome of induced sputum samples from 44 cigarette smoking asthma patients before and after attempting smoking cessation. Due to the longitudinal sampling design we could quantify the changes in the microbiome within each participant reducing inter-subject variations.

We found no significant difference in the changes in the bacterial communities between asthma patients who quit cigarette smoking and those who did not. However, as both groups attempted smoking cessation we cannot rule out that the patients in the non-quitter group changed their smoking habits in terms of the amount of daily use or intermittent quitting. Consequently, we investigated the change within the group of patients that successfully quit cigarette smoking and found no association between smoking cessation and the bacterial community. This suggests that there is no strong association between smoking cessation and changes in the sputum microbiome. Yet, it is still possible that a larger sample size or a longer follow-up period would have detected a change.

We did find that bacterial diversity, measured both by the number of unique OTUs and the Shannon index, was significantly higher in the cigarette smoking asthma patients compared to the healthy non-smoking controls. This corroborates previous findings showing that the bacterial diversity in the lungs of asthma patients is more diverse than that of healthy controls [6,8]. However, as the control-group consisted of non-smokers without asthma we cannot directly conclude on the association between asthma and the sputum microbiome.

In contrast to our and others findings [6,8], Park *et al*. have found that the bacterial diversity in oropharyngeal samples from asthma patients is lower than that of healthy controls [7]. However, as samples were collected from different sites, it is difficult to directly compare their findings with the results presented here.

At the bacterial family level we found two families to be significantly enriched in the sputum of cigarette smoking asthma patients compared to healthy controls; Streptococcaceae and Spirochaetaceae. Yet, it is difficult to evaluate the clinical implications of this finding as both families contain species commonly found in the oral cavity and upper respiratory tract. Furthermore, as we did not specify any hypothesis with respect to changes in specific bacterial families the analysis of the changes in bacterial families required correction for multiple hypotheses testing, which greatly reduces statistical power. As a result, it is difficult to interpret the non-significant findings with a low p-value in the families in Fig 3. Still, our exploratory analysis can aid in the hypothesis development for future studies of the bacterial communities in the lungs.

In the present study, samples were collected using induced sputum, which represents a non-invasive alternative to bronchoalveolar lavage (BAL). Samples were transferred to a LB + glycerol medium and immediately stored on ice. However, the nutrient rich medium could in theory have affected the bacterial population. In order to reduce oral contamination participants washed their mouth with water before giving the sputum sample. Yet, induced sputum samples will suffer from oral contamination.

Studies comparing the microbiome at different sites along the aerodigestive tract find that this body site constitutes a microbiological continuum with substantial species overlap but varying abundances, [4,21,23]. Consequently, it is challenging to define a distinct lung microbiome and to filter out upper airway- or oral contamination from the induced sputum 16S data.

Recently a study comparing the lung microbiome of healthy tobacco smokers and non-smokers found no difference between the two groups [4]. Since the study was done on healthy individuals, smoking associated changes in the lung microbiome that causes disease may be overlooked. Accordingly, some of the increased risk and severity of pulmonary disorders in tobacco smokers with asthma [11] could still be mediated through smoking-induced changes in the lung microbiome. However, our results suggest that the improvements in asthma control immediately following cigarette-smoking cessation is not directly linked with changes in the sputum microbiome.

## Supporting Information

S1 Fig Sequences per amplicon. The distribution of sequence reads per amplicon for the controls and the asthma patients at each sampling time.
(PDF)

S2 Fig OTUs per amplicon. The distribution of OTUs per amplicon for the controls and the asthma patients at each sampling time.
(PDF)

S3 Fig Sample rarefaction curves. Rarefaction curves per amplicon for the controls and the asthma patients at each sampling time.
(PDF)

S4 Fig Diversity at week 0 and week 12 stratified on randomization. The observed OTUs (A) and Shannon index (B) at week 0 and week 12 stratified by randomisation. When comparing the change in the observed OTU from week 0 to week 12 in the varenicline group to the change in the placebo group there is no significant difference (A) (p = 0.45, unpaired t-test of the within-patient change from week 0 to week 12 in the varenicline group compared to the within-patient change in the placebo group). Similarly, when comparing the change in Shannon index from week 0 to week 12 in the varenicline group to the change in the placebo group there is no significant difference (B) (p = 0.61, unpaired t-test of the within-patient change from week 0 to week 12 in the varenicline group compared to the within-patient change in the placebo group). The solid crossbars mark the mean of the distributions.
(PDF)

S5 Fig Comparison of quitters at week 0 to week 12. The observed OTUs (A) and Shannon index (B) of the quitters at week 0 and week 12. When comparing the observed OTU at week 0 to week 12 there is no significant difference (A) (p = 0.08, paired t-test). Similarly, when comparing the Shannon index at week 0 to week 12 there is no significant difference (B) (p = 0.38, paired t-test). The solid crossbars mark the mean of the distributions.
(PDF)
