# Supplementary material for: Smoking Cessation and the Microbiome in Induced Sputum Samples from Cigarette Smoking Asthma Patients
Source: PLoS One. 2016 Jul 8;11(7):e0158622. doi: 10.1371/journal.pone.0158622 (PMC4938234; doi:10.1371/journal.pone.0158622)
Supplement: S2 Fig — The distribution of OTUs per amplicon for the controls and the asthma patients at each sampling time. (PDF) [file pone.0158622.s002.pdf]

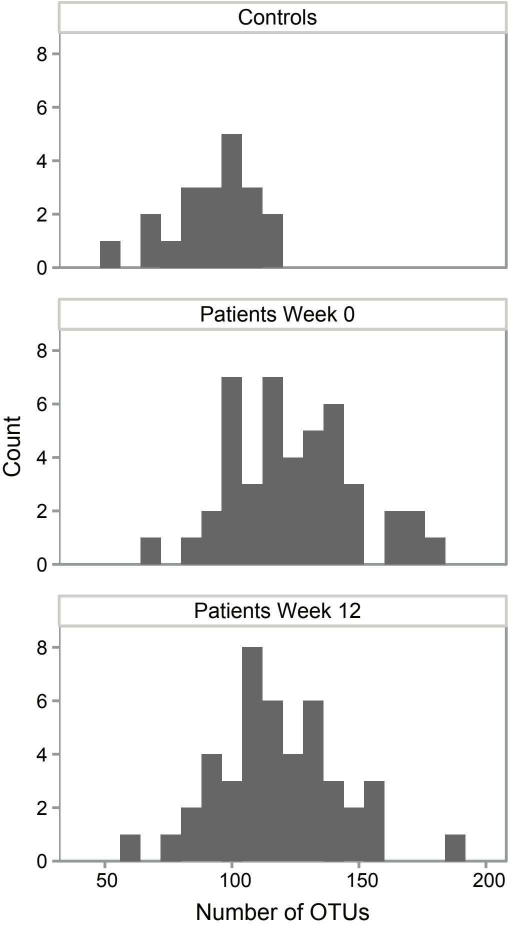

S2 Fig. OTUs per amplicon
The distribution of OTUs per amplicon for the controls and the asthma patients at each sampling time.
