# Supplementary material for: Smoking Cessation and the Microbiome in Induced Sputum Samples from Cigarette Smoking Asthma Patients
Source: PLoS One. 2016 Jul 8;11(7):e0158622. doi: 10.1371/journal.pone.0158622 (PMC4938234; doi:10.1371/journal.pone.0158622)
Supplement: S4 Fig — The observed OTUs (A) and Shannon index (B) at week 0 and week 12 stratified by randomisation. When comparing the change in the observed OTU from week 0 to week 12 in the varenicline group to the change in the placebo group there is no significant difference (A) (p = 0.45, unpaired t-test of the within-patient change from week 0 to week 12 in the varenicline group compared to the within-patient change in the placebo group). Similarly, when comparing the change in Shannon index from week 0 to week 12 in the varenicline group to the change in the placebo group there is no significant difference (B) (p = 0.61, unpaired t-test of the within-patient change from week 0 to week 12 in the varenicline group compared to the within-patient change in the placebo group). The solid crossbars mark the mean of the distributions. (PDF) [file pone.0158622.s004.pdf]

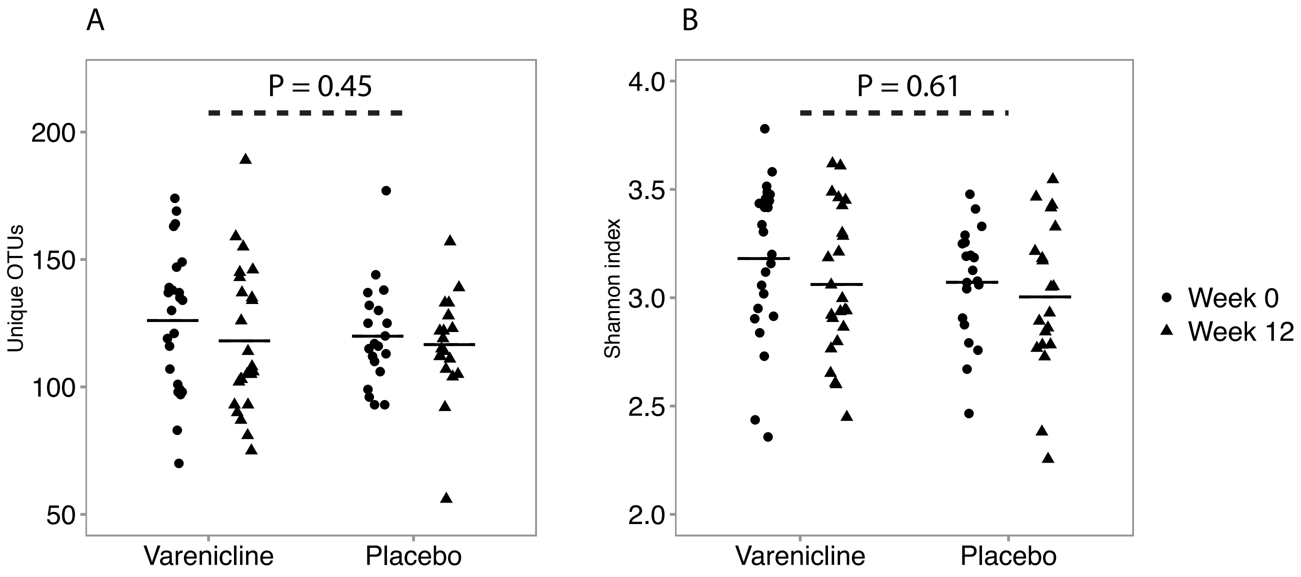

S4 Fig. Diversity at week 0 and week 12 stratified on randomization.
The observed OTUs (A) and Shannon index (B) at week 0 and week 12 stratified by randomisation. When comparing the change in the observed OTU from week 0 to week 12 in the varenicline group to the change in the placebo group there is no significant difference (A) ($p = 0.45$, unpaired t-test of the within-patient change from week 0 to week 12 in the varenicline group compared to the within-patient change in the placebo group). Similarly, when comparing the change in Shannon index from week 0 to week 12 in the varenicline group to the change in the placebo group there is no significant difference (B) ($p = 0.61$, unpaired t-test of the within-patient change from week 0 to week 12 in the varenicline group compared to the within-patient change in the placebo group). The solid crossbars mark the mean of the distributions.
