# Supplementary material for: Smoking Cessation and the Microbiome in Induced Sputum Samples from Cigarette Smoking Asthma Patients
Source: PLoS One. 2016 Jul 8;11(7):e0158622. doi: 10.1371/journal.pone.0158622 (PMC4938234; doi:10.1371/journal.pone.0158622)
Supplement: S5 Fig — The observed OTUs (A) and Shannon index (B) of the quitters at week 0 and week 12. When comparing the observed OTU at week 0 to week 12 there is no significant difference (A) (p = 0.08, paired t-test). Similarly, when comparing the Shannon index at week 0 to week 12 there is no significant difference (B) (p = 0.38, paired t-test). The solid crossbars mark the mean of the distributions. (PDF) [file pone.0158622.s005.pdf]

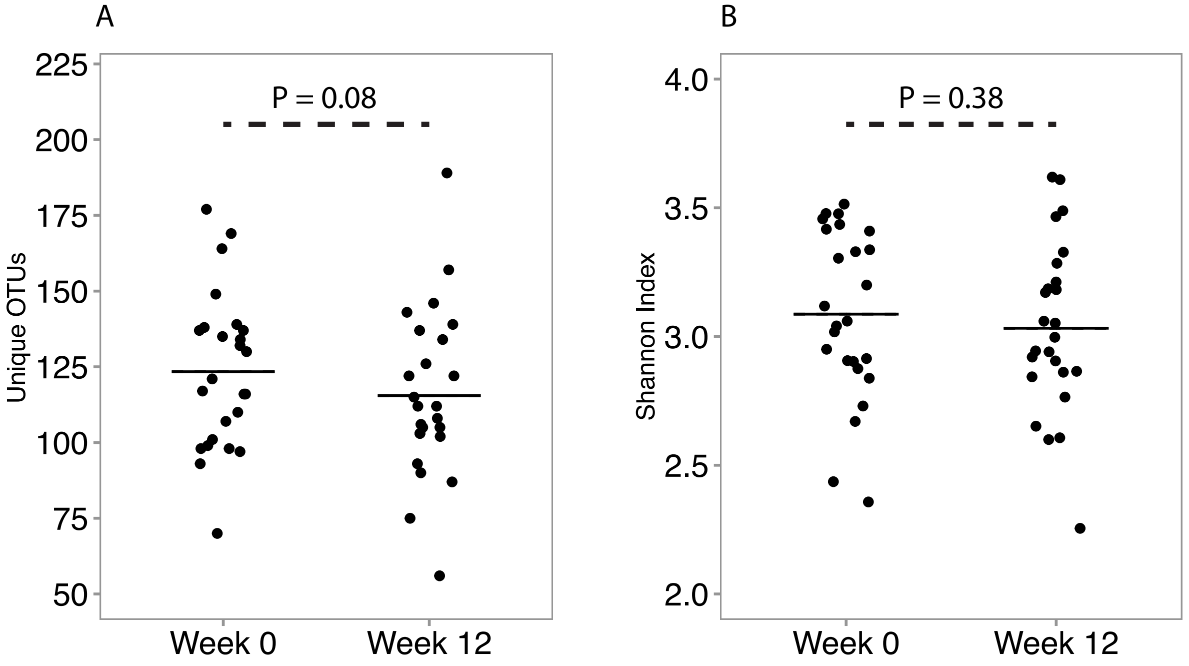

S5 Fig. Comparison of quitters at week 0 to week 12

The observed OTUs (A) and Shannon index (B) of the quitters at week 0 and week 12. When comparing the observed OTU at week 0 to week 12 there is no significant difference (A) (p = 0.08, paired t-test). Similarly, when comparing the Shannon index at week 0 to week 12 there is no significant difference (B) (p = 0.38, paired t-test). The solid crossbars mark the mean of the distributions.
